# miND (miRNA NGS Discovery pipeline): a small RNA-seq analysis pipeline and report generator for microRNA biomarker discovery studies

**DOI:** 10.12688/f1000research.94159.2

**Published:** 2026-05-09

**Authors:** Andreas Diendorfer, Kseniya Khamina, Marianne Pultar, Matthias Hackl

**Affiliations:** 1 TAmiRNA GmbH, Vienna, Austria

**Keywords:** microRNA, Next-Generation Sequencing, differential expression, smallRNA sequencing, biomarkers, spike-in, discovery study

## Abstract

In contrast to traditional methods like real-time polymerase chain reaction, next-generation sequencing (NGS), and especially small RNA-seq, enables the untargeted investigation of the whole small RNAome, including microRNAs (miRNAs) but also a multitude of other RNA species. With the promising application of small RNAs as biofluid-based biomarkers, small RNA-seq is the method of choice for an initial discovery study. However, the presentation of specific quality aspects of small RNA-seq data varies significantly between laboratories and is lacking a common (minimal) standard.

The miRNA NGS Discovery pipeline (miND) aims to bridge the gap between wet lab scientist and bioinformatics with an easy to setup configuration sheet and an automatically generated comprehensive report that contains all essential qualitative and quantitative results that should be reported. Besides the standard steps like preprocessing, mapping, visualization, and quantification of reads, the pipeline also incorporates differential expression analysis when given the appropriate information regarding sample groups.

Although miND has a focus on miRNAs, other RNA species like tRNAs, piRNA, snRNA, or snoRNA are included and mapping statistics are available for further analysis. miND has been developed and tested on a multitude of data sets with various RNA sources (tissue, plasma, extracellular vesicles, urine, etc.) and different species.

miND is a Snakemake based pipeline and thus incorporates all advantages using a flexible workflow management system. Reference databases are downloaded, prepared and built with an included (but separate) workflow and thus can easily be updated to the most recent version but also stored for reproducibility.

In conclusion, the miND pipeline aims to streamline the bioinformatics processing of small RNA-seq data by standardizing the processing from raw data to a final, comprehensive and reproducible report.

## Introduction

Small RNA-seq has been a well-established tool for the quantification of short RNA molecules like microRNAs (miRNAs) in various biofluids ( Murillo *et al*., 2019). Those short RNA molecules (17 to 25nt) play an important role in the cellular regulation of gene expression by interacting with specific complementary sites in targeted messenger RNAs (mRNAs). mRNAs that contain these target sites are then either down- or (rarely) up-regulated, resulting in a regulatory effect on the downstream translation of the mRNA ( O’Brien *et al*., 2018). In this context, miRNAs are part of a complex regulatory network where their expression does not only affect other mRNAs, but also the expression of miRNAs themselves is highly controlled ( Lee & Ambros, 2001). Thus, the levels of miRNAs can be indicators of a cell’s regulatory state and correlate with an organism’s health status. For example the liver specific miR-122-5p was shown to be a suitable marker for liver injury when measured in serum or plasma ( Llewellyn *et al*., 2021) and as part of a miRNA expression signature can even be used to predict recovery after liver resection ( Starlinger *et al*., 2019).

This makes them interesting targets as biomarkers in liquid biopsy ( Larrea *et al.*, 2016). The search for miRNAs or miRNA signatures suitable as biomarkers requires a specialized computational approach, and next-generation sequencing (NGS) is frequently used in the discovery phase of such studies ( de Ronde *et al.*, 2018). A number of tools for small RNA sequencing analysis are available, ranging from command-line tools such as miRDeep2 ( Friedländer *et al.*, 2012) and miRge 3.0 ( Patil & Halushka, 2021), to web-based platforms like Oasis 2.0 ( Rahman *et al.*, 2018) and sRNAbench ( Aparicio-Puerta *et al.*, 2022), and desktop applications such as the UEA sRNA Workbench ( Stocks *et al.*, 2018). These tools address different aspects of small RNA analysis, including read mapping, miRNA quantification, novel miRNA prediction, and in some cases differential expression. However, many focus on individual analysis steps and leave the integration of results and their presentation in an accessible format to the user.

To address the need for a standardized and integrated analysis workflow, we developed miND, a small RNA-seq processing pipeline that combines all steps from raw data to differential expression in a single reproducible workflow. The pipeline produces a comprehensive interactive HTML report designed to support interpretation by both bioinformaticians and biologists. Experimental metadata, including sample grouping and statistical contrasts, is provided through an Excel-based contrast sheet that serves as a structured interface between wet-lab scientists and bioinformaticians.

We developed a robust and portable analysis pipeline for small RNA NGS data with a focus on biomarker discovery, targeting three goals: (1) standardized data inputs, (2) reproducible analysis, and (3) accessible results for both bioinformaticians and study statisticians, including publication-ready figures and an intuitive representation of results.

The miND pipeline can be used on many operating systems and in various setups with the only requirement of being able to run Snakemake workflows ( Köster & Rahmann, 2012). Wrapper scripts for startup of the pipeline on Linux based systems are provided which can be adapted for the use on different platforms.

## Methods

### Implementation

The pipeline is based on Snakemake ( Köster & Rahmann, 2012), a scalable bioinformatics workflow engine which incorporates many features needed for reproducible computational analysis ( Mölder *et al*., 2021). This includes handling the installation and provisioning of software tools via conda ( “Anaconda Software Distribution,” 2020) and bioconda ( Grüning *et al*., 2018) and overall the orchestration of individual steps of the pipeline to optimize usage of limited resources like central processing unit (CPU) and memory. Configuration files in yml format are used and contain settings for multithreading to adapt the pipeline for various computing platforms ( Diendorfer *et al*., 2022).

## Use case

An example protocol demonstrating the analysis of a public data set is available at protocols.io under the name miND pipeline AWS EC2 installation and setup V.2 and can be reproduced not only as a guide for following data analysis, but also to setup the pipeline and data repository. The protocol describes the setup in an Amazon Web Services EC2 (Amazon Web Services, Inc, 2015) instance but has also been developed and tested on other platforms and systems. Only operating system specific parts would have to be adapted (e.g., installation of tools like git or wget would be done via apt on Debian based Linux distributions). For scientists interested in running the miND pipeline themselves, it is highly recommended to follow the provided protocol with the example data before running analysis on their own data sets.

The generated miND report for this example data set is available on GitHub.

### Operation

The miND pipeline was developed and tested on Debian Linux (v11.2) running Snakemake (v6.0.5) and conda (v.4.10.3). The hardware requirements depend on the size of analyzed datasets, but in general it is recommended to provide at least 4 CPU cores and 8GB of memory. The pipeline will scale according to the available resources.

### Data repository

The pipeline requires data from three reference data sets: (1) host genomes from ENSEMBL ( Zerbino *et al*., 2018), (2) RNA sequences from RNAcentral ( Sweeney *et al*., 2019), and (3) miRNA mature and precursor sequences from miRbase ( Griffiths-Jones, 2004).

In order to download and prepare these datasets in the formats and structures required, miND provides separate workflows to build the data repository. These workflows can be executed with a shell script that will read configurations for each data source and then download, format and build the reference databases based on Snakemake workflows.

The data repository only has to be built once and will then provide the data needed for all future miND analysis runs. In case of updates of reference data sets, the repository can be rebuilt or extended by adding sources to the configuration files and running the build script again.

### NGS raw data and metadata file

The miND pipeline requires two types of data for each experiment: raw NGS data and a meta data file with additional sample information. Raw data can be supplied either in fastq, fastq.gz or BAM (without alignments) files. The given format will be detected based on the file extensions.

Experimental meta data and details about the samples is provided in a XLS file containing three sheets: (1) Project details sheet, with general information and data of the project. This includes project title and comments but also settings relevant for the processing of the data like the sample species, adapter sequences, and cutoff levels for significance and quality filtering. (2) Sample group matrix sheet, which lists all samples that are part of this experiment and links them to additional group information. Up to five grouping variables can be set with unlimited levels each. The last sheet contains the (3) Contrast selection and allows the selection of groups and group-combinations based on the data provided in the sample group matrix sheet. The contrasts selected here will be used for the differential expression analysis.

### Pipeline analysis steps

**Figure 1.**
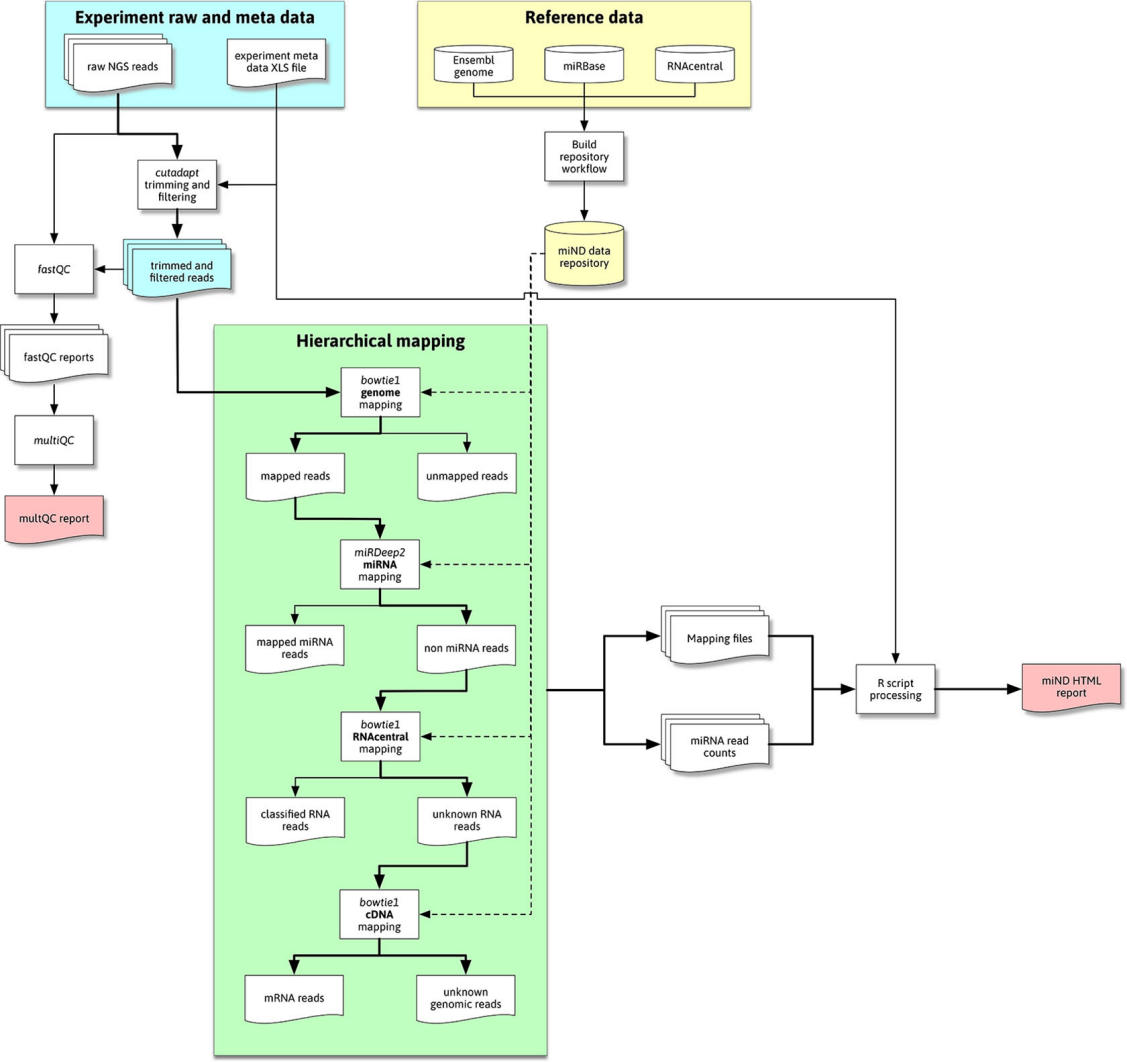
Flowchart representing the high-level steps of data processing through the pipeline. Reference data is downloaded and processed by the repository build process (yellow area; top right) and then available for the miND pipeline in the repository/subfolder. Raw next-generations sequencing (NGS) data (blue area) is first adapter and quality trimmed and then handled by quality control (QC) tools and processed through hierarchical mapping steps (green area). These steps produce a set of mapping files that are then ingested and analyzed by R scripts, producing the miND report in the end.

The overall flow of data through the pipeline is shown in Figure 1. This flow diagram outlines the most important steps of data processing in the miND pipeline, especially the quality control steps with FastQC ( Andrews, 2010) and multiQC ( Ewels *et al*., 2016), followed by hierarchical mapping using bowtie1 ( Langmead *et al*., 2009) and miRDeep2 ( Friedländer *et al*., 2012), where either mapped or unmapped reads are further processed by the next step. The final “R scripts processing” step includes multiple scripts that preprocess and analyze that data (including mapping statistics, unsupervised analysis methods and differential expression analysis) to then generate an interactive HTML report based on R markdown.

The hierarchical mapping uses genome datasets from the prepared data repository (generated once before the initial run as described in the “Data repository” subsection) in a first step to filter out reads that to not map to the host organism’s genome (bowtie1, allowing for two mismatches). The genome-mapped reads are further processed by miRDeep2 to accurately quantify miRNAs. To identify further remaining (genome mapping but non-miRNA) reads, bowtie1 is used to first map against the RNAcentral database and then complementary DNA sequences (to assign mRNA reads), both steps allowing for one mismatch. Reads that remain unmapped after these hierarchical clustering are classified as either “unknown genomic” (if they mapped against the host genome) or “unmapped” (in case of reads that did not map against the host genome and were thus filtered in the first mapping step). The generated mapping files are processed by R scripts to prepare mapping statistics for the different RNA species in each sample.

The mapping process focuses on miRNAs and prioritizes them by using the specialized mapping tool miRDeep2 directly after an initial genome mapping step. It utilizes bowtie1 for mapping of the reads but performs a more sophisticated assignment of miRNA IDs to the reads. This includes detailed information of isomiRs (mature miRNAs with highly similar sequences) that is prepared for further analysis steps.

For the identification of other RNA species RNAcentral is used. This comprehensive database contains non-coding RNA (ncRNA) sequences from a broad range of species. This step focuses on the classification of reads and uses bowtie1 (allowing for one mismatch) reporting the first (best) hit. This limits the use of the mapping data to the required classification, as reads could map to multiple references which are not reported mainly for performance reasons.

### Differential expression and independent filtering

miND pipeline uses the popular R package EdgeR ( Robinson *et al*., 2009) for differential expression analysis (DEA) with the quasi-likelihood negative binomial generalized log-linear model functions provided by the package.

A key step in differential expression analysis is the removal of lowly expressed features, which would otherwise increase noise and inflate false positive rates. Fixed RPM-based cutoff values (e.g., filtering miRNAs below 10 RPM) do not account for variation in library size and miRNA content and are therefore arbitrary. The DEA package DESeq2 ( Love *et al*., 2014) implements an independent filtering method that was adapted in miND to be used also with EdgeR. Assuming that most false-positives are caused by low abundant miRNAs, the algorithm removes quantiles of miRNAs from the low-abundance end and checks if the number of significant miRNAs increases after false-discovery rate (FDR) adjustment. This would be the case if mostly false positives have been removed because FDR adjustment would now be more sensitive and not remove as many true positives, increasing the overall number of significant results. This method works reliably when true positives are present. If no true positives exist, removing low-abundance miRNAs will not increase the number of significant results after FDR adjustment. For this case, the miND implementation includes a fallback: miRNAs with RPM values below 10 divided by the smallest library size, in at least half the samples of the smaller group, are pre-filtered before DEA and FDR adjustment. These miRNAs carry negligible biological and statistical relevance ( Chen *et al*., 2016).

**Figure 2.**
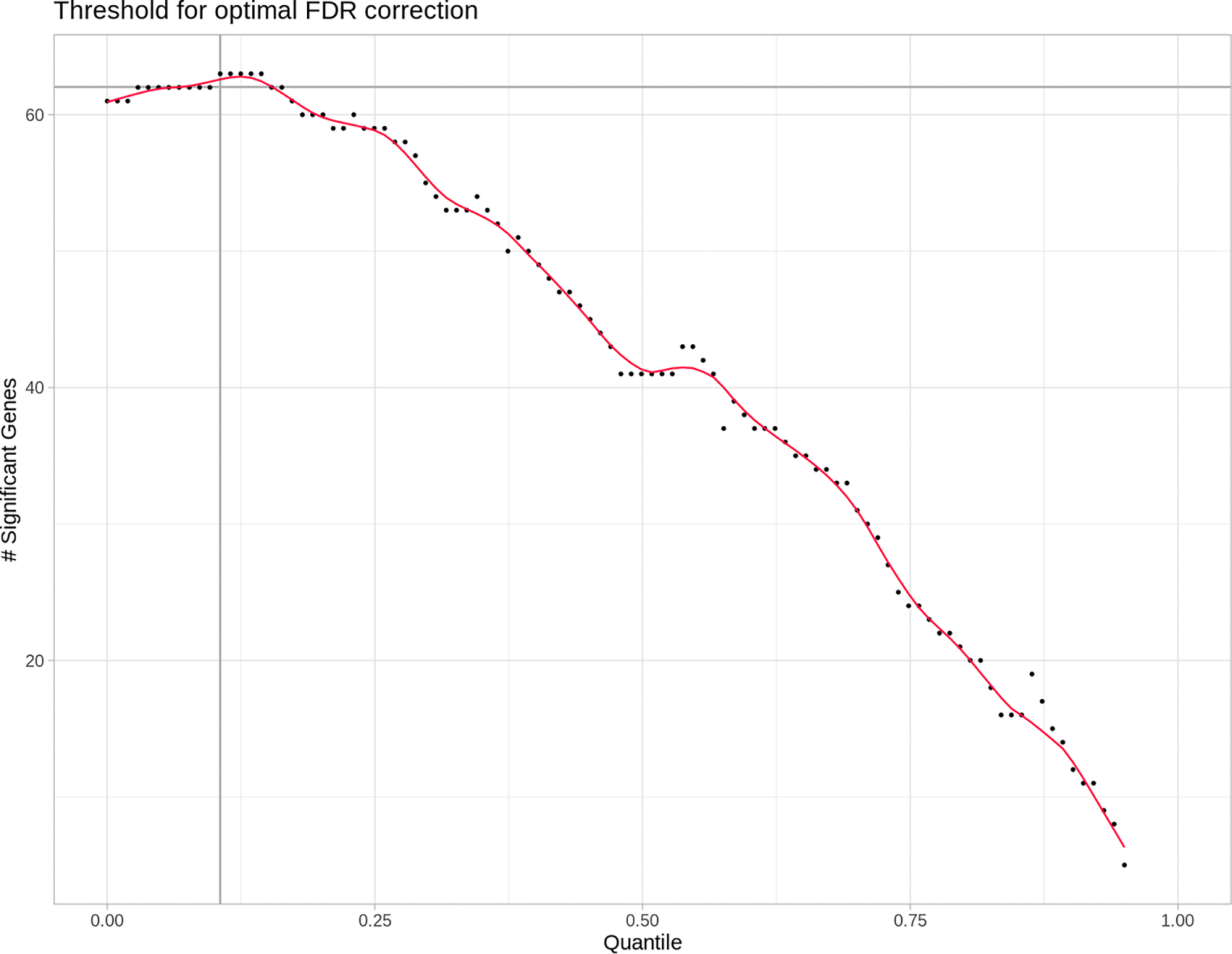
DESeq2's false discovery rate (FDR) based independent filtering method. Each point represents the number of differentially expressed micro ribonucleic acids (miRNAs) after false discovery rate (FDR) adjustment and done in steps of increasingly stringent quantile-based reads filtering. With more and more low read count miRNAs removed from the differential expression analysis, the number of significant (FDR) differentially expressed (DE) miRNAs increases to the point where more and more true positives get removed, thus decreasing the total amount of DE miRNAs. This is shown in the graph as the maximum of the red line. The optimal quantile cutoff value is then determined by finding this maximum.

An exemplary relation between a given quantile cut-off and the resulting number of differentially expressed miRNAs after FDR is shown in Figure 2.

For differential expression the contrasts of interest can be selected in the experiment meta data XLS file (last sheet of the SampleContrastSheet.xlsx). Either groups or combinations of groups can be selected based on the group information provided for the samples. Each selected contrast will be part of the final interactive HTML report. In addition, a blocking factor can be selected if applicable. This blocking factor will be included in the model for the differential expression as additive factor and thus can be used e.g., for a paired experimental design or to account for batch effects.

### Interactive HTML report and statistical analysis

**Figure 3.**
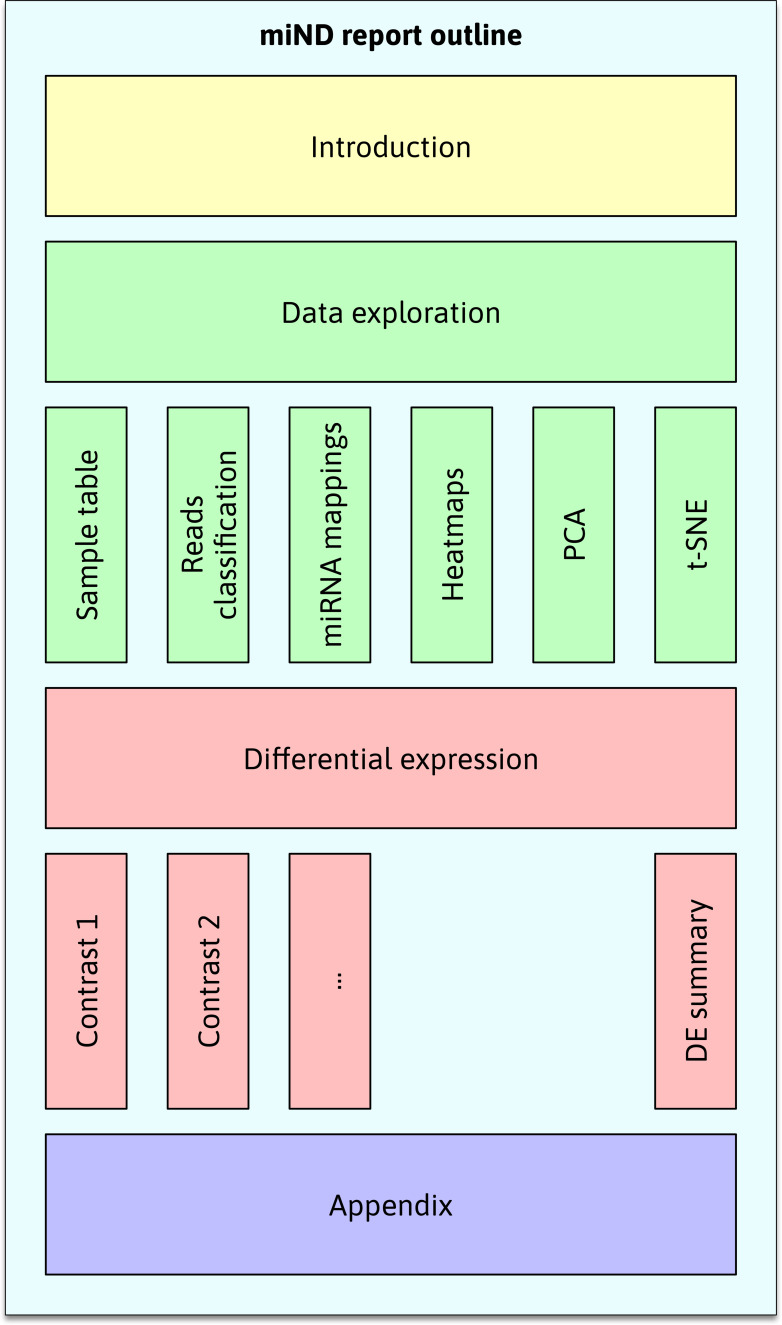
Outline of the interactive miND report. The main sections (1) introduction, (2) data exploration, (3) differential expression, and (4) appendix each contain multiple subsections. The standardized structure of the report allows for the quick assessment and comparison of experiment results. t-distributed stochastic neighbor embedding (t-SNE), micro ribonucleic acids (miRNAs), differentially expressed (DE).

Although DEA is a central point of biomarker discovery studies, other statistical methods are needed to put this analysis into context and ensure valid results. The miND pipeline report contains a series of additional graphs and tables to present the data in a way that is interactive and easy to browse. The main sections (see Figure 3) are (1) introduction, (2) data exploration (including a sample table, reads classification plots, miRNA mapping tables, heatmaps, principal component analysis (PCA) and t-distributed stochastic neighbor embedding (t-SNE plots)), (3) differential expression results, and (4) an appendix (references and run information).

*Reads classification plots*

**Figure 4.**
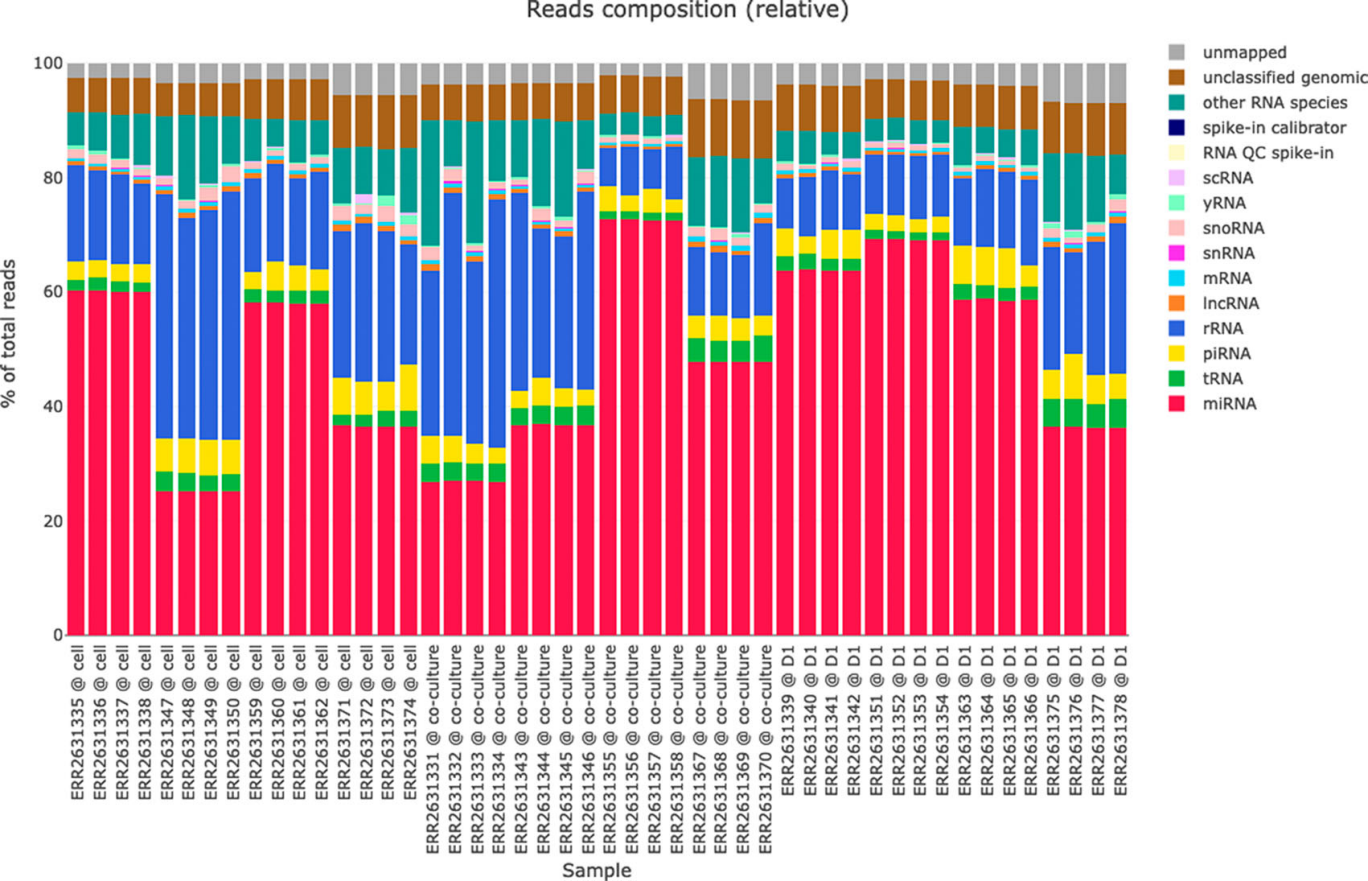
Reads classification of all samples scaled to 100% of total reads. Each bar represents an individual sample, while colors of the bar charts give insights in the mapped ribonucleic acid (RNA) species. This representation helps with a quick identification of library prep or sequencing issues if the ratios or total number of reads are not as expected.

The reads classification plots (see Figure 4) present the amounts of reads mapped to different RNA species (miRNAs, tRNAs, piRNA, rRNA, lncRNA, etc.) based on the hierarchical mapping done by the miND pipeline. This is plotted as absolute reads but also as relative ratios (percent) to get a quick impression of the RNA classes that are present in the data set. Especially for samples with low numbers of miRNAs present (e.g. extracellular vesicles) these two graphs give important information about the success of library preparation and sequencing. While the differential expression analysis in miND focuses on miRNAs, the quantification and visualization of other small RNA species in the reads classification provides a useful overview of the small RNA composition in each sample.

The data on which the reads classification plots are based on is also included in the HTML report and can be either browsed directly in the HTML file or (as all tables and figures) or exported in various data formats (CSV or XLS for tabular data and PNG for graphics) for further analysis or publications.

*miRNA mappings table*

The miRNA mappings table contains read counts for each miRNA that was found in at least one of the samples. The table is available with raw read counts but also as RPM (normalized to the total number of miRNAs mapped in each sample). Group information is included in this table, if provided by the experiment metadata XLS file.

**Figure 5.**
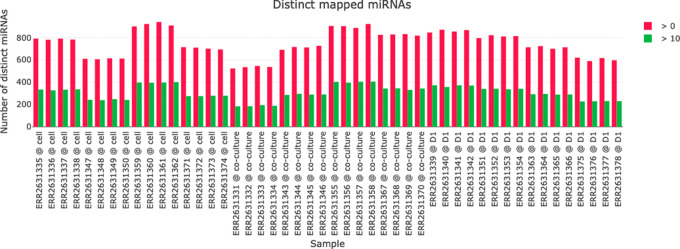
Distinct mapped micro ribonucleic acids (miRNAs) for each sample. The number of identified miRNAs with either a read count above 0 (red) or 10 (green) is plotted for each sample.

A visualization of the miRNA mapping statistics helps in comparing the number if identified miRNAs in the samples (see Figure 5). For each sample the number of distinct miRNAs with a read count above 0 and above 10 is plotted to give an impression about the abundance of distinct miRNAs and their read counts in the samples.

*Heatmaps, PCA and t-SNE plots*

**Figure 6.**
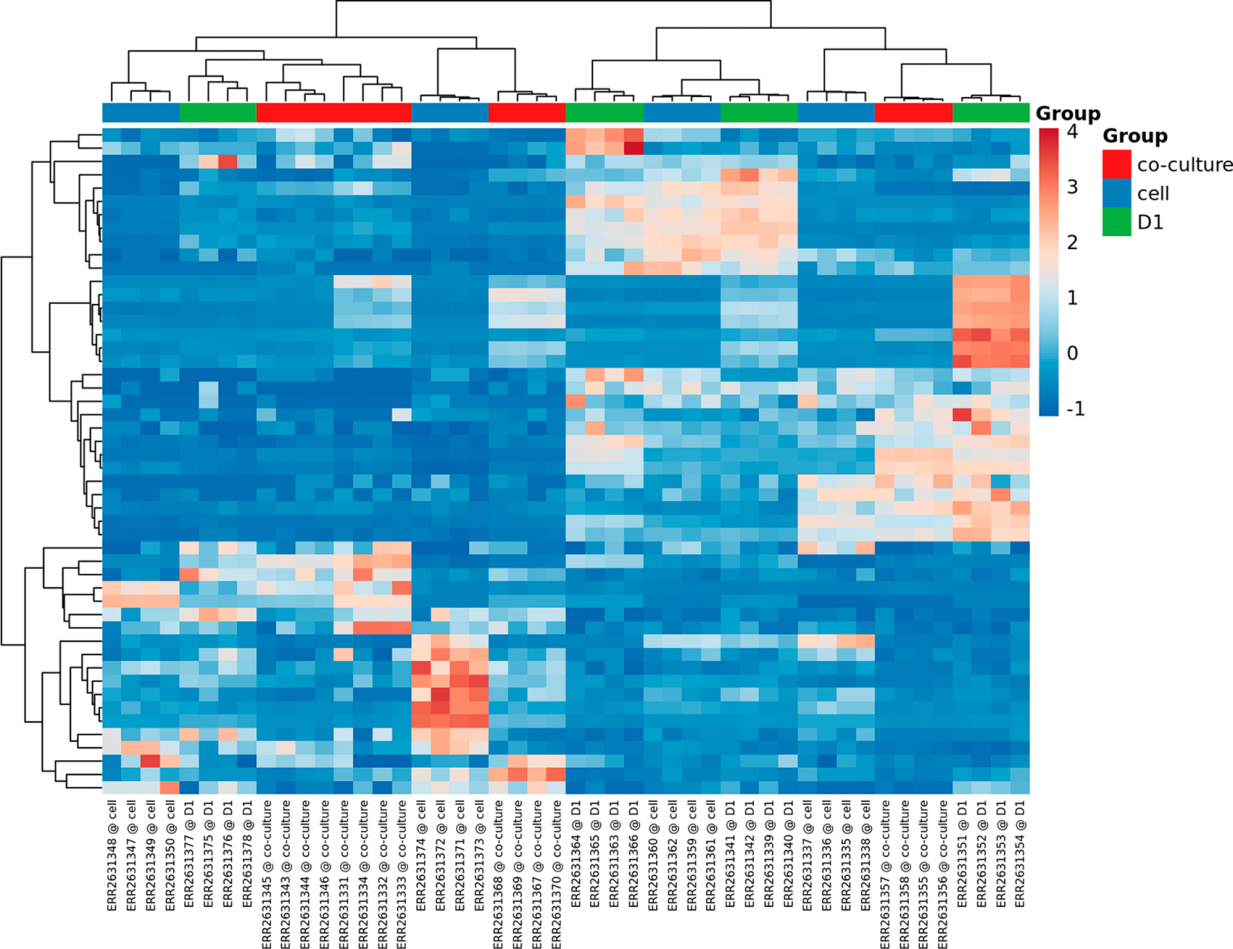
Heatmap of top 50 miRNAs. Group information provided with the experiment meta data XLS file is included if available.

The heatmaps, PCA and t-SNE plots are part of the unsupervised clustering methods that are applied by the miND pipeline and included in the report. For better understanding of underlaying group relationships, any grouping information available in the meta data file will be included in the graphs. Two heatmaps are generated in the interactive HTML report. The first includes only the top 50 miRNAs based on the coefficient of variation (see Figure 6) while the second one contains all miRNAs that were detected in all samples. Both heatmaps are based on RPM normalized reads and scaled using the unit variance method for visualization. Clustering is based on complete clusters using Euclidean distances as these methods are applicable for most experimental setups. The group association of each sample is shown in the heatmaps with colored bars at the top to visualize clustering of samples based on the provided grouping information. Multiple groups are supported for heatmaps (no groups limit) and PCA/t-SNE (maximum of two groups are shown by colors and shapes).

## Conclusions

The miND pipeline was developed and optimized for miRNA-focused analysis of small RNA-seq data, with particular emphasis on biomarker discovery studies. While other available tools focus on specific aspects of the analysis (e.g., miRDeep2 on quantification of miRNAs and annotation of possible novel miRNAs and sRNAbench on differential expression), miND generates an extensive and standardized report suitable for the discovery phase of biomarker studies. The prepared HTML report provides a solid basis for further research and communicates the most important results in a structured and accessible way. Especially parameters relevant to quality control of the whole sequencing experiment (from library preparation to the in-silico analysis) are reported in standardized formats, to allow for a reliable and quick analysis of the overall quality of the experiment.

Besides the results, the generated HTML report includes descriptions, hints, and details about the methods used. This ensures that the results can be interpreted and understood easily by non-statisticians or bioinformaticians. In addition, it ensures that the final HTML report contains all information needed for reproducibility and documentation of the analysis.

Data input and experimental setup of the miND pipeline can be adjusted with the given meta data file, making it possible to use the miND pipeline for various species, sample matrices and library preparation protocols.

With the availability of the source code of the pipeline under the GNU General Public License, additional analysis steps can be integrated into the R markdown report if needed, allowing the pipeline to be tailored to other specialized applications.

While miND provides an extensive set of analyses for early-phase biomarker discovery, no standardized pipeline can cover every study’s specific requirements. The results generated are meant to be a starting point for further analysis and optimizations, as parameters. For example, differential expression or heatmaps are chosen to give good results in most use cases but might not be the optimal for an individual project.

*Comparison with existing tools*

Several tools for small RNA sequencing analysis have been published in recent years, each with different strengths and design goals ( Table 1). Some focus on specific steps of the analysis, such as miRDeep2, which provides miRNA quantification and novel miRNA prediction through a probabilistic hairpin model. miND builds directly on miRDeep2 for these tasks and extends its output into a complete analysis workflow that includes differential expression analysis and a structured interactive report.

**Table 1.** Feature comparison of small RNA-seq analysis tools. miRDeep2 is included as the foundational miRNA quantification tool integrated within the miND pipeline.

Feature	miND	miRDeep2	miRge 3.0	Oasis 2.0	sRNAbench	Prost!	sRNAPipe	sRNAWorkbench
**Reference**	Diendorfer et al., 2022	Friedländer et al., 2012	Patil & Halushka, 2021	Rahman et al., 2018	Aparicio-Puerta et al., 2022	Desvignes et al., 2019	Pogorelcnik et al., 2018	Stocks et al., 2018
**Runtime environment**	Snakemake, conda (Linux CLI)	Perl (Linux CLI)	Python CLI; Electron GUI available	Web platform	Web server; standalone JAR; Docker	Python 2.7 CLI (+ BBMap/Java)	Galaxy	Java desktop application (GUI + CLI)
**Raw data processing**	QC, adapter trimming, size and quality filtering (FASTQ, FASTQ.gz, BAM)	Adapter clipping, read collapsing	Adapter trimming via Cutadapt (FASTQ)	QC, adapter trimming (FASTQ)	QC, adapter trimming (FASTQ, SRA, Google Drive, Dropbox)	No; expects pre-trimmed FASTA input	No; expects pre-trimmed FASTQ input	Adapter removal, filtering (built-in)
**Sample metadata input**	Excel sheet with sample groups and contrast selection for DE analysis	Config file (sample ID mapping)	CSV file with group assignment	Web form for groups and covariates	Web form; Excel/text annotation for DE grouping (sRNAde)	Text file mapping filenames to sample names	Via Galaxy interface	Via GUI workflow
**miRNA quantification**	miRDeep2 (bowtie1)	bowtie1 with hairpin-aware assignment	bowtie1	bowtie (via miRDeep2)	bowtie1	BBMap against user-defined annotation	BWA	PatMaN/bowtie (via miRProf)
**Other ncRNA quantification**	RNAcentral mapping (tRNA, piRNA, rRNA, snRNA, snoRNA, lncRNA, yRNA)	No	tRNA fragments, snoRNA, rRNA, mRNA	snRNA, snoRNA, rRNA, piRNA	tRNA, snoRNA, snRNA, rRNA, yRNA; custom annotation upload	Depends on user-provided annotation	tRNA, rRNA, snRNA, gene transcripts, transposable elements; piRNA/siRNA by size class	miRNA, siRNA, ta-siRNA
**Novel miRNA prediction**	Yes (via adaptions and integrated miRDeep2)	Yes (core feature; Bayesian scoring model)	Yes (SVM-based)	Yes (via miRDeep2)	Yes (random forest classifier)	No (genome-first approach facilitates manual discovery)	No	Yes (miRCat2)
**Differential expression**	edgeR with adapted independent filtering	No	DESeq2 (optional, built-in)	DESeq2	DESeq, DESeq2, edgeR, NOISeq (via sRNAde module)	No	No	Yes (custom LOFC method with multiple normalizations)
**Summary report**	Comprehensive interactive HTML report with interpretation guidance and data export	HTML overview with PDF hairpin structure plots	Interactive HTML report with charts and tables	Web dashboard; downloadable interactive HTML reports	Individual result files; interactive web summaries	Excel workbook (7 sheets)	HTML report with plots, count tables, BAM/bedgraph files	Interactive visualizations via GUI
**Open source, self-hosted**	Yes (GPL-3.0)	Yes (GPL-3.0)	Yes (MIT)	No (web service only)	Yes (MIT)	Academic license (non-commercial use)	Yes (AFL-3.0)	Yes (MIT)

Other tools offer broader analysis capabilities through different interfaces. Oasis 2.0 ( Rahman *et al.*, 2018) provides an accessible web-based platform that requires no local installation, making it straightforward to use for researchers without command-line experience. sRNAbench ( Aparicio-Puerta *et al.*, 2022) is available both as a web service and a standalone application and offers one of the most comprehensive sets of analysis modules, including multiple methods for differential expression via its sRNAde module. The UEA sRNA Workbench ( Stocks *et al.*, 2018) provides a Java desktop application with tools for miRNA discovery and differential expression, originally developed for plant small RNA research but now supporting both plant and animal datasets. sRNAPipe ( Pogorelcnik *et al.*, 2018) integrates small RNA analysis into the Galaxy platform and includes specialized support for piRNA analysis including ping-pong signature detection. Prost! ( Desvignes *et al.*, 2019) focuses on accurate read quantification against user-defined annotations and is well suited for organisms with limited existing small RNA annotation. miRge 3.0 ( Patil & Halushka, 2021) provides fast miRNA profiling with support for tRNA fragment analysis and UMI-based deduplication.

miND is specifically designed for miRNA biomarker discovery studies. Its distinguishing feature is the combination of structured metadata input through an Excel-based contrast sheet, an integrated Snakemake workflow that handles all processing steps from raw FASTQ files to differential expression results, and a comprehensive interactive HTML report. The contrast sheet allows biologists to define sample groups and statistical comparisons in a familiar spreadsheet format, while the HTML report presents quality control metrics, RNA class distributions, unsupervised clustering, and differential expression results in a single document with interactive elements and export functions for publication-ready figures. This design supports collaboration between wet-lab scientists and bioinformaticians: while biologists prepare the metadata and interpret the report, bioinformaticians handle pipeline execution.

The miND pipeline was developed as part of the Translational Safety Biomarker Pipeline (TransBioLine) project from the IMI2 consortium. This project focuses on the discovery of miRNAs as novel biomarkers in the context of drug safety. In this case, the miND pipeline provides a standardized but still extensive first analysis of NGS data. In addition, the miND pipeline includes an extra module for the implementation of miND spike-ins for absolute quantification of microRNAs as recently published by Khamina *et al*. (2022).

In another recently published article by Gutmann *et al*. (2021) the pipeline was used in the discovery phase of the study to identify miRNAs that are associated with COVID-19 severity and mortality. The miRNAs reported by the miND pipeline were later manually selected and evaluated based on the HTML report for further confirmation with RT-qPCR, where the confirmation showed a high level of reproducibility from the NGS data.

We will continue working on the pipeline and release updates to the public version if needed. Especially in regard to the miND spike-ins that allow for the absolute quantification of miRNA in biofluids we expect to release an updated version soon.

### Limitations

The execution of the miND pipeline requires bioinformatics expertise, including familiarity with the Linux command line. No graphical user interface or web-based access is provided. However, the contrast sheet and interactive HTML report are designed to be accessible without bioinformatics training, so that the preparation of experimental metadata and the interpretation of results can be handled by biologists directly.

The differential expression analysis in miND is focused on miRNAs. Other small RNA species (including tRNAs, piRNAs, rRNAs, snRNAs, snoRNAs, and lncRNAs) are quantified through RNAcentral mapping and their distributions are visualized in the report, but they are not included in the differential expression analysis. It has to be noted, that the mapping against other RNA species is not as specific as the miRNA mapping, as miRNAs are mapped early on in the pipeline to allow for a targeted mapping, while RNAcentral mapping is done in one step which can lead to inaccuracies for reads mapping to multiple targets and reference databases inside RNAcentral. This reflects the pipeline’s focus on miRNA biomarker discovery. The quantification framework could be adapted to support differential expression of other small RNA species in the future.

miRNA annotation relies on miRBase as the reference database. Results are therefore dependent on the completeness and accuracy of miRBase entries for the organism under study. Finally, the hardware requirements for running miND are modest: small RNA sequencing datasets are typically compact, and analysis of dozens of samples is feasible on standard desktop hardware with four or more CPU cores and 8 GB of memory.

## Data availability

### Source data

Mature and hairpin sequences of miRBase are available at: https://www.mirbase.org/ftp/22.1

Genome sequences (DNA and cDNA) is available at Ensembl (for human): http://ftp.ensembl.org/pub/release-105/fasta/homo_sapiens

Non-coding RNA sequences are available at RNAcentral: http://ftp.ebi.ac.uk/pub/databases/RNAcentral/current_release

Data associated with the example use case are not owned by the authors. Requirements to access these datasets is given in the protocol ( https://dx.doi.org/10.17504/protocols.io.b3f6qjre).

## Software availability

Source code available from: https://github.com/tamirna/miND

Archived source code available from: https://doi.org/10.5281/zenodo.6080470 ( Diendorfer *et al., *2022)

License: GNU GPL 3.0
