# Supplementary material for: miND (miRNA NGS Discovery pipeline): a small RNA-seq analysis pipeline and report generator for microRNA biomarker discovery studies
Source: F1000Res. 2026 May 9;11:233. Originally published 2022 Feb 24. [Version 2] doi: 10.12688/f1000research.94159.2 (PMC13173284; doi:10.12688/f1000research.94159.2)
Supplement: Peer review history

# Reviewer report: Reviewer response for version 2

**DOI:** 10.5256/f1000research.198035.r483661

**Published:** 2026-05-14

**Authors:** Iddo Magen

**Affiliations:** 1 Weizmann Institute of Science, Rehovot, Israel

The authors explained clearly the pipeline and its features, with proper visualizations and diagrams, and included reference databases and comparison to previous pipelines. They addressed very well the reviewers' concerns. I do not have any specific comments for this manuscript and I think it is ready for indexing. This pipeline can be used easily by researchers in the field.

Are the conclusions about the tool and its performance adequately supported by the findings presented in the article?

Yes

Is the rationale for developing the new software tool clearly explained?

Yes

Is the description of the software tool technically sound?

Yes

Are sufficient details of the code, methods and analysis (if applicable) provided to allow replication of the software development and its use by others?

Yes

Is sufficient information provided to allow interpretation of the expected output datasets and any results generated using the tool?

Yes

Reviewer Expertise:

biomarkers in neurodegeneration.

I confirm that I have read this submission and believe that I have an appropriate level of expertise to confirm that it is of an acceptable scientific standard.

# Reviewer report: Reviewer response for version 2

**DOI:** 10.5256/f1000research.198035.r483190

**Published:** 2026-05-13

**Authors:** Kristian Almstrup, Ailsa Maria Main, Nina Mørup

**Affiliations:** 1 Department of Growth and Reproduction, Copenhagen University Hospital - Rigshospitalet, Copenhagen, Denmark

The revision has clearly improved the paper, and the comparison with other similar tools makes it of higher value to the readers.

Are the conclusions about the tool and its performance adequately supported by the findings presented in the article?

Partly

Is the rationale for developing the new software tool clearly explained?

Partly

Is the description of the software tool technically sound?

Yes

Are sufficient details of the code, methods and analysis (if applicable) provided to allow replication of the software development and its use by others?

Yes

Is sufficient information provided to allow interpretation of the expected output datasets and any results generated using the tool?

Yes

Reviewer Expertise:

Endocrinology, small RNAs, genetics

We confirm that we have read this submission and believe that we have an appropriate level of expertise to confirm that it is of an acceptable scientific standard.

# Reviewer report: Reviewer response for version 2

**DOI:** 10.5256/f1000research.198035.r483191

**Published:** 2026-05-13

**Authors:** Francisco J. Enguita

**Affiliations:** 1 Faculdade de Medicina, Universidade de Lisboa, Lisboa, Portugal

Dear Editor

 I have checked the answers and modifications introduced in the manuscript by the Authors, and I sincerely think that they have substantially improved the text. I would recommend it for publication.

 Sincerely

Are the conclusions about the tool and its performance adequately supported by the findings presented in the article?

No

Is the rationale for developing the new software tool clearly explained?

Partly

Is the description of the software tool technically sound?

Partly

Are sufficient details of the code, methods and analysis (if applicable) provided to allow replication of the software development and its use by others?

Partly

Is sufficient information provided to allow interpretation of the expected output datasets and any results generated using the tool?

Partly

Reviewer Expertise:

non-coding RNAs; miRNAs; lncRNAs; circRNAs; structural biology

I confirm that I have read this submission and believe that I have an appropriate level of expertise to confirm that it is of an acceptable scientific standard.

# Reviewer report: Reviewer response for version 1

**DOI:** 10.5256/f1000research.101122.r151316

**Published:** 2022-10-05

**Authors:** Francisco J. Enguita

**Affiliations:** 1 Faculdade de Medicina, Universidade de Lisboa, Lisboa, Portugal

The manuscript by Diendorfer and coworkers describes a pipeline for NGS data processing specially devoted to the analysis of small non-coding RNAs, mainly focused on miRNAs.

 The manuscript is well written, but the authors would need to give further details in order to compare their pipeline with the already existing ones. It is not clear for the reader what are the main advantages of miND pipeline in comparison with the already available ones.

 I would advise to perform a small benchmarking study using a test dataset that could be any one existing in public databases. The authors would need to answer the questions: what are the advantages of miND, its weaknesses and why the user should give a try to this new pipeline.

Are the conclusions about the tool and its performance adequately supported by the findings presented in the article?

No

Is the rationale for developing the new software tool clearly explained?

Partly

Is the description of the software tool technically sound?

Partly

Are sufficient details of the code, methods and analysis (if applicable) provided to allow replication of the software development and its use by others?

Partly

Is sufficient information provided to allow interpretation of the expected output datasets and any results generated using the tool?

Partly

Reviewer Expertise:

non-coding RNAs; miRNAs; lncRNAs; circRNAs; structural biology

I confirm that I have read this submission and believe that I have an appropriate level of expertise to confirm that it is of an acceptable scientific standard, however I have significant reservations, as outlined above.

# Response

**Published:** 2026-03-23

**Authors:** Andreas B. Diendorfer

**Affiliations:** TAmiRNA GmbH, Austria

We thank both reviewers for their constructive comments and suggestions. We have revised the manuscript to address all points raised. Below we provide a point-by-point response. All changes are marked in the revised manuscript.

**Comment 1: Comparison with existing pipelines**

*"The authors would need to give further details in order to compare their pipeline with the already existing ones. It is not clear for the reader what are the main advantages of miND pipeline in comparison with the already available ones."*

**Response**: We have added a feature comparison table that systematically compares miND against seven other small RNA analysis tools. We have also expanded the Discussion to position miND in the context of these tools and clearly articulate its design goals and distinguishing features: the combination of an Excel-based contrast sheet for metadata input, an integrated Snakemake workflow building on miRDeep2, and a comprehensive interactive HTML report tailored for biomarker discovery studies.

**Comment 2: Benchmarking study**

*"I would advise to perform a small benchmarking study using a test dataset that could be any one existing in public databases."*

**Response**: Rather than a data-driven benchmark, we have provided a feature-level comparison that we believe is more informative for readers selecting a tool. The choice of a small RNA analysis pipeline typically depends on the required analysis scope, interface preferences, and reporting needs. The manuscript already demonstrates miND's output using a public dataset (PRJEB27261/E-MTAB-6885), and the generated example report is available on GitHub.

**Comment 3: Advantages, weaknesses, and rationale**

*"The authors would need to answer the questions: what are the advantages of miND, its weaknesses and why the user should give a try to this new pipeline."*

**Response**: We have addressed this through three changes: (1) the expanded Introduction now provides a broader context of existing tools, (2) the new "Comparison with existing tools" paragraphs in the Discussion articulate miND's specific strengths, and (3) the new Limitations paragraph honestly discusses its constraints. miND's primary value lies in its end-to-end integration from raw data to an interactive report, the structured contrast sheet that facilitates collaboration between biologists and bioinformaticians, and its focus on the specific needs of miRNA biomarker discovery studies.

# Reviewer report: Reviewer response for version 1

**DOI:** 10.5256/f1000research.101122.r129126

**Published:** 2022-04-14

**Authors:** Kristian Almstrup, Nina Mørup, Ailsa Maria Main

**Affiliations:** 1 Department of Growth and Reproduction, Copenhagen University Hospital - Rigshospitalet, Copenhagen, Denmark

In the manuscript by Diendorfer et al., a bioinformatic pipeline for analysis of data from small RNA sequencing is presented. The pipeline, named miND, allows identification and annotation of small RNA reads as well as differential expression analysis.

 I have the following major concerns about the study as it is:

 Several other small RNA sequencing pipelines, like Oasis2.0 ( https://oasis.dzne.de/index.php), sRNAWorkbench ( https://sourceforge.net/projects/srnaworkbench/), sRNAPipe ( https://github.com/GReD-Clermont/sRNAPipe), miRge3.0 ( https://sourceforge.net/projects/mirge3/) already exist and some support both identification and differential expression analysis. It is hence unclear what novelty miND brings compared to other pipelines. To allow the reader to make an informed choice about which pipeline to choose for analysis, miND should be benchmarked against some of the already existing pipelines. What are the differences when the same dataset (PRJEB27261/E-MTAB-6885) is analysed with e.g. Oasis2.0 (Rahman *et al.,* 2018)?

 The authors argue that miND “bridges the gap between biologists and bioinformaticians”, and this is also evident from the easy-to-use Excel files. However, the pipeline is based on Snakemake workflows and a conda install and hence require *a priori* knowledge of conda, which would not be common knowledge to biologists. I encourage the authors to make miND available as a standalone app or web portal (as is the case for the similar pipeline Oasis2.0). Since, at least, parts of the miND pipeline are based on R-scripts it might be easy to make a Shiny app or similar.

 The pipeline focuses on miRNAs. This reviewer encourages the authors also to include analysis of other small RNA species as these are likely to be equally important as biomarkers in a liquid biopsy. Furthermore, on a whole, the authors do not discuss in detail the possible problems and downsides of their pipeline. A more in-depth and critical discussion of strengths and limitations is warranted.

 The readme file on github should contain instructions on how to install miND in a language that biologists can understand.

 Finally, in some places the authors should consider shortening sentences/simplifying statements so, again, it is easier for the readers to follow.

Are the conclusions about the tool and its performance adequately supported by the findings presented in the article?

Partly

Is the rationale for developing the new software tool clearly explained?

Partly

Is the description of the software tool technically sound?

Yes

Are sufficient details of the code, methods and analysis (if applicable) provided to allow replication of the software development and its use by others?

Yes

Is sufficient information provided to allow interpretation of the expected output datasets and any results generated using the tool?

Yes

Reviewer Expertise:

Endocrinology, small RNAs, genetics

We confirm that we have read this submission and believe that we have an appropriate level of expertise to confirm that it is of an acceptable scientific standard, however we have significant reservations, as outlined above.

## References

[1] : Oasis 2: improved online analysis of small RNA-seq data. *BMC Bioinformatics* .2018;19(1) : 10.1186/s12859-018-2047-z 10.1186/s12859-018-2047-z 29444641 PMC5813365

# Response

**Published:** 2026-03-23

**Authors:** Andreas B. Diendorfer

**Affiliations:** TAmiRNA GmbH, Austria

We thank both reviewers for their constructive comments and suggestions. We have revised the manuscript to address all points raised. Below we provide a point-by-point response. All changes are marked in the revised manuscript.

**Comment 1: Benchmarking against existing pipelines**

*"Several other small RNA sequencing pipelines, like Oasis2.0, sRNAWorkbench, sRNAPipe, miRge3.0 already exist and some support both identification and differential expression analysis. It is hence unclear what novelty miND brings compared to other pipelines. To allow the reader to make an informed choice about which pipeline to choose for analysis, miND should be benchmarked against some of the already existing pipelines."*

**Response**: We agree that a systematic comparison with existing tools is valuable and have added a feature comparison table to the manuscript. The table covers miND, miRDeep2, miRge 3.0, Oasis 2.0, sRNAbench, Prost!, sRNAPipe, and sRNAWorkbench across dimensions including runtime environment, raw data processing, metadata input, miRNA and other ncRNA quantification, novel miRNA prediction, differential expression analysis, summary reporting, and licensing. We note that miRDeep2 is included as the foundational quantification tool that miND builds upon, rather than as a competing pipeline.

 Rather than a data-driven benchmark on a single dataset, which would primarily reflect differences in read mapping and counting strategies, we opted for a feature-level comparison. We believe this is more useful for readers choosing a tool, as the choice typically depends on the required analysis scope, interface, and reporting capabilities rather than marginal differences in miRNA detection rates.

 We have also expanded the Discussion to contextualize miND's positioning among these tools (see revised Discussion section "Comparison with existing tools").

**Comment 2: Web portal / standalone app / Shiny app**

*"The pipeline is based on Snakemake workflows and a conda install and hence require a priori knowledge of conda, which would not be common knowledge to biologists. I encourage the authors to make miND available as a standalone app or web portal."*

**Response**: We appreciate this suggestion. miND is intentionally designed as a self-hosted, open-source pipeline. This ensures that users and institutions are not dependent on third-party infrastructure, which can become unavailable when hosting organizations discontinue support. The open-source license (GPL-3.0) enables any institution to deploy the pipeline freely.

 We would like to clarify that miND does not aim to be directly operated by biologists without bioinformatics support. The contrast sheet and interactive HTML report are designed to serve as the interface between wet-lab scientists and bioinformaticians: biologists prepare the experimental metadata in a familiar Excel format and interpret the report, while bioinformaticians handle pipeline installation and execution. We have revised the text to make this distinction clearer.

 To lower the barrier for bioinformaticians setting up the pipeline, we have rewritten the README on GitHub with step-by-step installation instructions and usage guidance. A detailed setup protocol is also available on protocols.io (doi: 10.17504/protocols.io.b3f6qjre).

**Comment 3: Other small RNA species**

*"The pipeline focuses on miRNAs. This reviewer encourages the authors also to include analysis of other small RNA species as these are likely to be equally important as biomarkers in a liquid biopsy."*

**Response**: miND does quantify other small RNA species. Through RNAcentral mapping, the pipeline classifies reads into multiple RNA categories including tRNAs, piRNAs, rRNAs, snRNAs, snoRNAs, and lncRNAs. These distributions are visualized in the reads classification plots of the HTML report. However, the differential expression analysis is focused on miRNAs, reflecting the pipeline's primary use case in miRNA biomarker discovery. The quantification framework could be adapted to support DE analysis of other small RNA species in the future.

 We have clarified this in the revised manuscript (see reads classification section and the new Limitations paragraph in the Discussion).

**Comment 4: Discussion of problems and downsides**

*"The authors do not discuss in detail the possible problems and downsides of their pipeline. A more in-depth and critical discussion of strengths and limitations is warranted."*

**Response**: We have added a dedicated Limitations paragraph to the Discussion section. It addresses the requirement for bioinformatics expertise to run the pipeline, the miRNA-centric scope of the DE analysis, the dependency on miRBase for miRNA annotation, and the hardware requirements.

**Comment 5: README on GitHub**

*"The readme file on github should contain instructions on how to install miND in a language that biologists can understand."*

**Response**: We have rewritten the README on GitHub (https://github.com/TAmiRNA/miND) with a description of the pipeline, its features, hardware requirements, installation steps, usage instructions, and output descriptions. The README also references the detailed protocols.io protocol for step-by-step setup guidance.

**Comment 6: Sentence length and readability**

*"In some places the authors should consider shortening sentences/simplifying statements so, again, it is easier for the readers to follow."*

**Response**: We have revised the manuscript for clarity throughout, shortening long sentences and simplifying phrasing where possible. Specific changes include the Introduction (paragraph on existing tools), the independent filtering method description, and parts of the Conclusions.
